# Carrageenan Is a Potent Inhibitor of Papillomavirus Infection

**DOI:** 10.1371/journal.ppat.0020069

**Published:** 2006-07-14

**Authors:** Christopher B Buck, Cynthia D Thompson, Jeffrey N Roberts, Martin Müller, Douglas R Lowy, John T Schiller

**Affiliations:** 1 Laboratory of Cellular Oncology, National Cancer Institute, Bethesda, Maryland, United States of America; 2 Forschungsschwerpunkt für Angewandte Tumorvirologie, Deutsches Krebsforschungszentrum, Heidelberg, Germany; University of Wisconsin, United States of America

## Abstract

Certain sexually transmitted human papillomavirus (HPV) types are causally associated with the development of cervical cancer. Our recent development of high-titer HPV pseudoviruses has made it possible to perform high-throughput in vitro screens to identify HPV infection inhibitors. Comparison of a variety of compounds revealed that carrageenan, a type of sulfated polysaccharide extracted from red algae, is an extremely potent infection inhibitor for a broad range of sexually transmitted HPVs. Although carrageenan can inhibit herpes simplex viruses and some strains of HIV in vitro, genital HPVs are about a thousand-fold more susceptible, with 50% inhibitory doses in the low ng/ml range. Carrageenan acts primarily by preventing the binding of HPV virions to cells. This finding is consistent with the fact that carrageenan resembles heparan sulfate, an HPV cell-attachment factor. However, carrageenan is three orders of magnitude more potent than heparin, a form of cell-free heparan sulfate that has been regarded as a highly effective model HPV inhibitor. Carrageenan can also block HPV infection through a second, postattachment heparan sulfate–independent effect. Carrageenan is in widespread commercial use as a thickener in a variety of cosmetic and food products, ranging from sexual lubricants to infant feeding formulas. Some of these products block HPV infectivity in vitro, even when diluted a million-fold. Clinical trials are needed to determine whether carrageenan-based products are effective as topical microbicides against genital HPVs.

## Synopsis

Sexually transmitted human papillomavirus (HPV) infections are very common. Although most HPV infections don't cause noticeable symptoms, persistent infection with some genital HPV types can lead to cervical cancer or other anal/genital cancers. Another subset of HPV types can cause genital warts. Recent studies have suggested that condoms are not highly effective in preventing HPV infection. Although HPV vaccines will soon become available, they probably will not protect against all genital HPV types and will be too expensive for use in the developing world. Inexpensive HPV-inhibitory compounds (known as topical microbicides) might be useful for blocking the spread of HPV. Using a newly developed cell culture–based HPV inhibition test, we have discovered that an inexpensive gelling agent called carrageenan is an unexpectedly potent HPV infection inhibitor. Carrageenan is also under investigation as a topical microbicide targeting HIV and herpes viruses, but it is a thousand times more effective against HPV in cell culture tests. Interestingly, carrageenan is used as a thickener in some commercially available sexual lubricants and lubricated condoms. Several of these commercial lubricant products are potent HPV inhibitors in our cell culture–infection system. Clinical trials are needed to determine the effectiveness of carrageenan as a topical microbicide against HPV.

## Introduction

Papillomaviruses are a diverse group of nonenveloped DNA viruses that infect the skin and mucosal tissues of a range of vertebrate species, including humans. A group of genital mucosotropic human papillomavirus (HPV) types are etiologic agents responsible for virtually all cases of cancer of the uterine cervix, as well as a substantial fraction of other ano-genital and head-and-neck cancers (reviewed in [1]). Cancer-associated genital HPV types, as well as another subset of HPV types associated with the development of benign genital warts (condyloma accuminata), are generally transmitted through sexual contact. Infection with genital HPV types is very common, with an estimated lifetime risk of infection of about 75% [2]. Although most genital HPV infections are subclinical and self-limiting, a subset of persistently infected individuals have lesions that progress to premalignancy or cancer.

Recent meta-analyses have suggested that condoms are, at best, only marginally effective for preventing the sexual transmission of HPV [3,4]. However, a highly effective group of prophylactic HPV vaccines are expected to become publicly available in the near future [5]. Two possible drawbacks to these vaccines are that they are expected to be relatively expensive (at least initially) and are likely to be papillomavirus type-restricted in their protection. Thus, the vaccines may not initially be available to women in all parts of the world and may not offer protection against all cancer-associated HPV types. Inexpensive condom-compatible compounds that could function as broad-spectrum topical microbicides targeting sexually transmitted HPVs might therefore serve as useful adjuncts to vaccination programs.

In vitro analysis of papillomavirus infection has historically been hampered by the fact that key events in the late phase of the viral lifecycle, such as the expression of the capsid proteins L1 and L2, require cellular differentiation in the upper layers of the stratified squamous epithelial tissues that the viruses inhabit (reviewed in [6]). As a consequence, papillomaviruses cannot replicate in conventional monolayer cell cultures. Investigation of the assembly and entry phases of the papillomavirus lifecycle has recently been simplified by the development of high-yield methods for producing papillomavirus-based gene transfer vectors, known as pseudoviruses (PsV), using conventional monolayer cell lines [7,8]. We have used PsV to develop a high-throughput screening method to identify and compare compounds with the potential to block papillomavirus infectivity in vitro [9].

Previous studies have shown that sulfated polysaccharides, such as heparin, cellulose sulfate, and dextran sulfate, can block the infectivity of papillomaviruses [10–12]. For many classes of virus, including papillomaviruses, initial attachment of the virion to cultured cell lines is thought to be mediated mainly by interactions between the virion and a type of cell-surface glycosaminoglycan (GAG) known as heparan sulfate (reviewed in [13]). In general, sulfated polysaccharides are thought to block viral infection by chemically mimicking heparan sulfate, thereby competing against initial virion attachment to the cell surface. Many previous studies have used various types of heparin, a highly sulfated form of heparan sulfate produced by mast cells, as a model inhibitor of papillomavirus infectivity. In this report we show that carrageenan, a class of sulfated polysaccharide extracted from marine red algae (seaweed), inhibits the infectivity of genital HPV PsV in vitro with nearly a thousand-fold greater potency than heparin.

Carrageenan is in widespread commercial use as a thickening agent for a variety of foods and cosmetic products, including some brands of sexual lubricant. Since high-quality carrageenan preparations (reviewed in [14]) appear to have a good safety profile for long-term vaginal use [15,16], carrageenan might be useful as an inexpensive topical microbicide for blocking the sexual transmission of HPV.

## Results

### Testing of Candidate Microbicides, including Carrageenan

Various candidate HPV infection inhibitors were tested using a PsV-based inhibition assay [9]. The assay uses flow cytometric analysis to assess the inhibition of PsV-mediated delivery of a green fluorescent protein (GFP) reporter plasmid into HeLa cells. HPV16, an exceptionally oncogenic genital HPV type [17], was chosen as a model HPV for initial experiments.

A wide variety of compounds were screened using the inhibition assay (Tables 1 and 2). Although a variety of nonsulfated polysaccharides failed to inhibit the infectivity of the HPV16 PsV, most types of sulfated polysaccharides we tested were inhibitory (Table 2). A standard heparin preparation was inhibitory at doses similar to previous reports [11,18]. However, preparations of intestine- and kidney-derived heparan sulfate displayed no detectable inhibition of PsV infectivity (Table 2). Since heparan sulfate modification is complex and varies depending on tissue source (reviewed in [13]), it is possible that the noninhibitory heparan sulfate preparations simply lack the proper chemical features (e.g., a particular pattern of sulfation) required to inhibit infection.

**Table 1:**
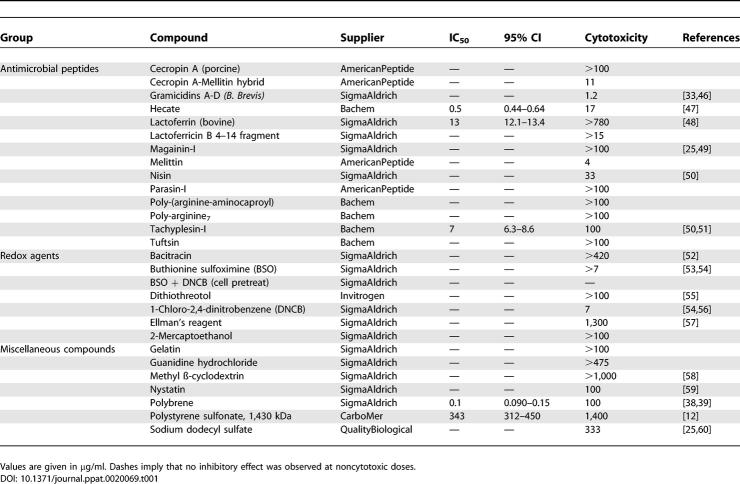
Inhibitory Effects of Nonpolysaccharide Compounds

**Table 2:**
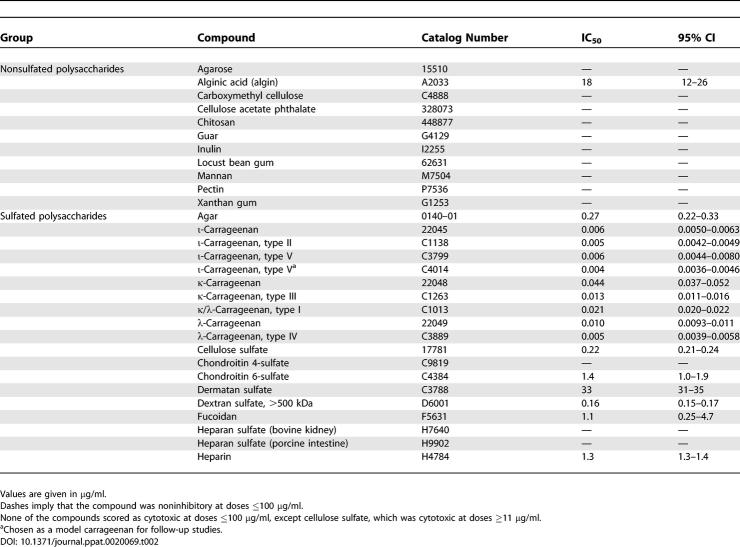
Inhibitory Effects of Polysaccharide Compounds

**Figure 1:**
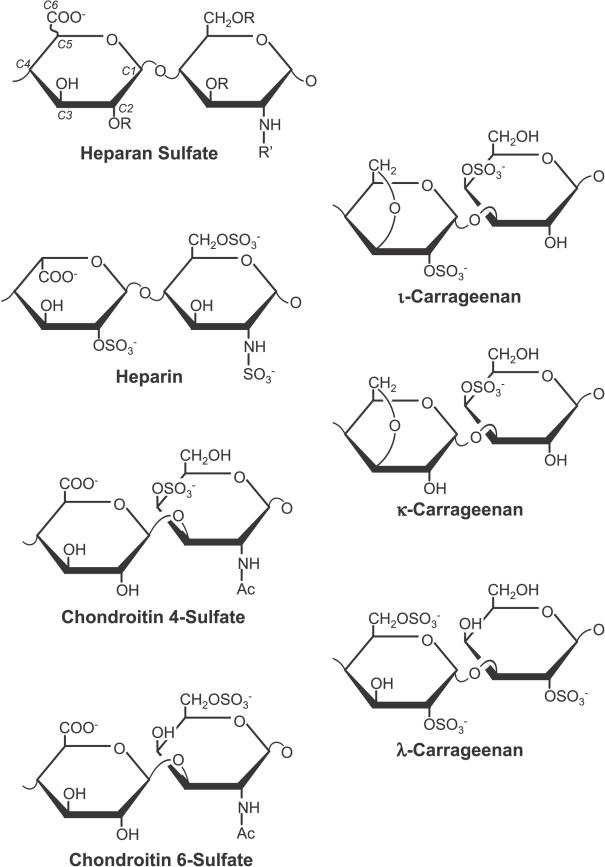
Polysaccharide Structures Idealized average structures of the disaccharide repeat units of various sulfated polysaccharides. Ac = acetyl, R = [H or SO_3_^−^], R′ = [H, Ac, or SO_3_^−^]. Structures adapted from [13], http://www.lsbu.ac.uk/water/hycar.html, and http://www.sigmaaldrich.com.

GAGs are unbranched polysaccharides primarily composed of a monotonous series of characteristic disaccharide repeats. GAGs can be divided into two broad categories: glucosaminoglycans, such as heparan sulfate, and galactosaminoglycans, such as chondroitin sulfate. As their names imply, a defining difference between the two GAG classes is their initial incorporation of *N*-acetyl-glucosamine or *N*-acetyl-galactosamine saccharide units, respectively. Another important difference between the two classes of GAG is that glucosaminoglycans are linked in a series of 1,4 saccharide bonds, whereas galactosaminoglycans are linked in an alternating series of 1,3 and 1,4 bonds. Average structures for examples of both types of GAG are shown in Figure 1.

Despite the substantial chemical differences between the average structures of chondroitin 6-sulfate and heparin, preparations of the two polysaccharides displayed similar inhibitory effects against the HPV16 PsV (Table 2). In contrast, a chondroitin 4-sulfate preparation was noninhibitory (Table 2 and [11]), despite its high degree of chemical similarity to chondroitin 6-sulfate. The results are consistent with a previous report demonstrating that chondroitin 6-sulfate (but not chondroitin 4-sulfate) competes against the interaction of noninfectious capsids with immobilized heparin [10].

Various types of carrageenan were by far the most potent inhibitors identified in the screen (Table 2). Carrageenan is an unbranched polysaccharide composed of galactose derivatives arranged in an alternating series of 1,3 and 1,4 saccharide linkages reminiscent of the pattern seen in galactosaminoglycans (Figure 1). In addition to having a similar pattern of saccharide linkages, the typical sulfation pattern of κ-type carrageenan also closely resembles chondroitin 4-sulfate, with an average of one 4-O-linked sulfate group per disaccharide repeat in both types of polysaccharide (Figure 1). Despite these apparent chemical similarities to the noninhibitory chondroitin 4-sulfate, κ-carrageenan was an extremely potent inhibitor, with 50% inhibitory concentration (IC_50_) values in the low ng/ml range.

Like heparin, λ- and ι-carrageenan types are more heavily sulfated than most tissue-derived heparan sulfate (reviewed in [13]). On average, these carrageenan types exhibited somewhat greater inhibitory potency than κ-carrageenan (Table 2).

### The Influence of Capsid Dose on Carrageenan Inhibitory Effects

**Figure 2:**
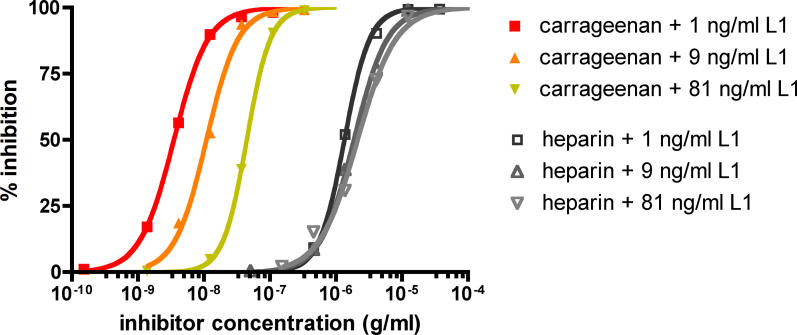
Capsid Dose Influences Carrageenan IC_50_ Inhibition assays were performed using a standard dose of GFP-expressing HPV16 PsV in the presence of increasing doses of cold capsids. Overall capsid dose is given as the final concentration of the major capsid protein, L1, in the culture medium.

Assuming a typical carrageenan chain length of about a thousand saccharide units (reviewed in [14]), the roughly 5 ng/ml IC_50_ of ι-carrageenan preparations corresponds to a concentration of about 20 pM. The standard PsV-based inhibition assay uses an HPV16 capsid inoculum of 1 ng of the major capsid protein, L1, per milliliter (also ~20 pM). Thus, the observed IC_50_s for the various types of carrageenan occurred under conditions where there was only a slight mass (or molar) excess of carrageenan over L1. If the inhibitory effects of carrageenan were due to its direct binding to the capsid, the IC_50_ might be expected to shift if excess capsids were added to the assay. Consistent with this hypothesis, the addition of “cold” capsids (i.e., PsV produced in the absence of a GFP reporter plasmid) resulted in a dose-dependent reduction in the apparent inhibitory potency of ι-carrageenan preparation C4014 (Figure 2). This was not true for the inhibitory effects of heparin, presumably because IC_50_s for heparin occur under conditions of substantial inhibitor excess, thus satisfying the law of mass action.

**Figure 3:**
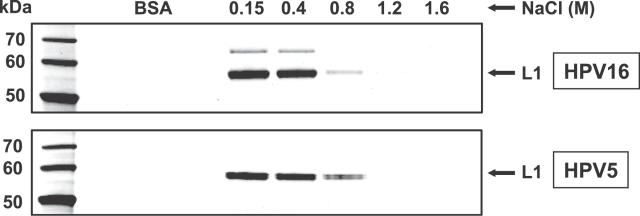
Capsids Bind Carrageenan Carrageenan beads were incubated with HPV16 or HPV5 capsids in buffers with the NaCl concentration shown. The beads were washed, then bound capsids were eluted and visualized in stained SDS-PAGE gels. Bovine serum albumin (BSA, 65 kDa) was used as a control for nonspecific binding to the beads. For HPV16, an L2 band can be seen above the L1 band.

The observation that excess capsids can increase the IC_50_ for carrageenan strongly implies that the primary inhibitory mechanism involves the direct binding of carrageenan to the capsid. To further investigate this hypothesis, we performed capsid pull-down experiments using ι-carrageenan beads. The results show that purified HPV16 capsids directly bind carrageenan in phosphate buffer containing ≤ 0.4 M NaCl (Figure 3, top panel).

Taken together, the results suggest the possibility that carbohydrate-binding motifs on the capsid surface are highly selective for a limited subset of sulfated polysaccharide sequences. Although it is tempting to speculate that the ι-carrageenan structure depicted in Figure 1 represents an ideal (or near-ideal) binding substrate, it should be noted that the biochemistry of GAG and carrageenan modification is complex, and preparations of these molecules can contain localized sequences of alternatively modified saccharide residues that differ substantially from the idealized structures depicted in Figure 1. Thus, the possibility that inhibition of the PsV is mediated by a subset of atypical saccharide sequences cannot be ruled out.

### Inhibitory Effects of ι-Carrageenan against Various Papillomavirus Types

The effectiveness of sulfated polysaccharides for blocking the in vitro infectivity of other viruses, such as HIV-1 and dengue viruses, varies dramatically according to which viral strain is used [19–21]. Thus, it seemed possible that the inhibitory effects of carrageenan might also vary for different papillomavirus types.

**Figure 4:**
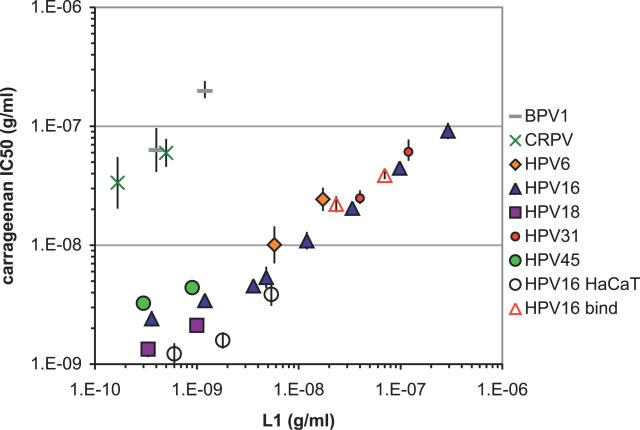
Standardized Carrageenan IC_50_ for Various Papillomavirus Types Points represent carrageenan IC_50_ of infectivity, except for empty red triangles, which represent the carrageenan IC_50_ of cell binding for HPV16 capsids covalently linked to a fluorescent dye. Empty circles represent carrageenan IC_50_ of infectivity observed using HaCaT cells instead of HeLa cells. Error bars represent the 95% CI for the IC_50_.

A variety of papillomavirus types are available as PsV. The particle-to-infectivity ratios for stocks of different PsV types vary substantially. We therefore used HPV16 cold capsids to generate a standard curve comparing the IC_50_ of ι-carrageenan inhibition to total capsid dose (Figure 4). Compared to this standard curve, PsV based on three other cancer-associated genital HPV types, 18, 31, and 45, exhibited a similar degree of susceptibility to inhibition by ι-carrageenan. PsV based on HPV6, a relatively nononcogenic type that can cause genital warts, also showed similar susceptibility to ι-carrageenan when compared to the HPV16 PsV.

Papillomaviruses are divided phylogenetically into more than a dozen genera, with genital HPV types occupying a single genus, alpha [22]. PsV are currently available for papillomaviruses from three other genera: delta (bovine papillomavirus type 1 [BPV1]), kappa (cottontail rabbit papillomavirus [CRPV]) and beta (HPV5). In contrast to the genital HPVs, PsV based on BPV1 and CRPV, which cause nongenital skin lesions in host animals, were about a hundred-fold less susceptible to inhibition by carrageenan (Figure 4).

Surprisingly, HPV5, which typically infects nongenital skin without causing overt symptoms, was not inhibited by ι-carrageenan or heparin, even at doses of up to 100 μg/ml. Despite this extreme resistance to inhibition by carrageenan, HPV5 PsV were found to bind directly to ι-carrageenan beads (Figure 3, bottom panel).

The same hierarchy of inhibition was seen for the various papillomavirus types when the assay was performed using other cell lines, including the spontaneously immortalized human keratinocyte line HaCaT, human 293TT cells, or murine C127 cells (Figure 4 and unpublished data).

To verify that ι-carrageenan is active against an authentic papillomavirus, we performed assays examining the focal transformation of C127 cells by BPV1 [23,24]. These experiments confirmed that ι-carrageenan can inhibit the infectivity of an authentic papillomavirus with an IC_50_ of between 1 and 10 μg/ml (unpublished data), consistent with capsid dose-adjusted IC_50_s observed for BPV1 PsV.

### The Influence of pH on Carrageenan Inhibitory Effects

Since carrageenan might have utility as a topical microbicide for preventing the sexual transmission of HPVs, it is important to consider the fact that human vaginal pH is typically below 4.5 (reviewed in [25]). We therefore performed inhibition assays examining the inhibitory effects of ι-carrageenan in culture medium buffered to pH 4.5 or 5.0 with lactic or acetic acid, respectively. ι-Carrageenan remained effective for blocking infectivity under acidic conditions, with IC_50_ 95% confidence intervals (CIs) of 17–20, 13–16, or 2.4–6.1 ng/ml at pH 4.5, 5.0, and 7.4, respectively.

### ι-Carrageenan Can Block Postbinding Events

To investigate the concept that ι-carrageenan might block infectivity by preventing the initial attachment of capsids to cells, we performed flow cytometric analysis of cells exposed to fluorescently labeled HPV16 capsids in the presence of various doses of carrageenan. As expected, ι-carrageenan blocked the binding of labeled capsids at concentrations similar to those capable of blocking infectivity when standardized for capsid dose (Figure 4). Similar results were obtained using HaCaT cells as a binding target (unpublished data).

**Figure 5:**
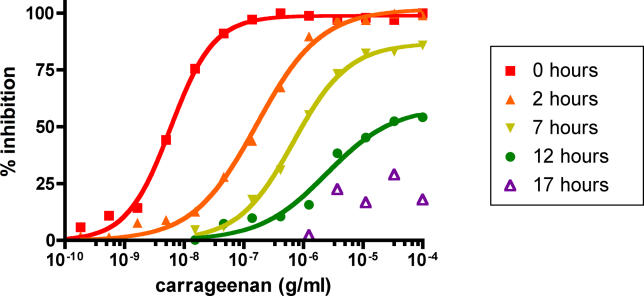
Carrageenan Addition Time Course Cells were incubated with HPV16 PsV for 2 h, followed by washout of the virus inoculum. Carrageenan was added at the timepoints shown, where time zero represents initial PsV inoculation.

In addition to blocking the initial attachment of virions to cells, heparin can also block the infectivity of cell-bound PsV for many hours after initial attachment to cells [11]. To investigate the possibility that ι-carrageenan also exerts additional, postattachment inhibitory effects on PsV infectivity, we performed time course experiments in which cells with prebound PsV were exposed to ι-carrageenan. As seen in the 2-h timepoint in Figure 5, cell-bound HPV16 PsV remained entirely susceptible to inhibition by somewhat higher doses of ι-carrageenan. Half of the infectious titer remained susceptible to inhibition by ι-carrageenan for up to 12 h after initial binding to cells. Similar results were observed when HaCaT cells were used as an infection target (unpublished data). The results demonstrate that, in addition to blocking the initial interaction of capsids with cells, ι-carrageenan also exerts a postattachment inhibitory effect on infectivity.

### Postbinding Inhibitory Effects of ι-carrageenan Do Not Involve Heparan Sulfate

The postattachment inhibitory effects of ι-carrageenan could be due either to displacement of capsids from heparan sulfate proteoglycans (HSPGs), or due to disruption of HSPG-independent steps in the viral infectious pathway. To address this issue, we made use of a GAG-negative cell line known as pgsA-745. The line was created by chemical mutagenesis of Chinese hamster ovary (CHO) cells, resulting in the disruption of a xylosyltransferase gene that is required for the first peptide glycosylation step in the synthesis of all GAGs, including heparan sulfate [26]. Fluorescent capsid binding studies (unpublished data) confirmed a previous report showing that pgsA-745 cells bind HPV capsids relatively poorly compared to parental CHO cells [10]. Despite this reduction in bulk capsid binding, pgsA-745 cells could be infected to the same extent as parental CHO cells if a 50-fold higher inoculum of HPV16 PsV was used (data not shown).

Using these conditions, we examined the effects of ι-carrageenan on HPV16 PsV prebound to the two cell lines for 3 h. Both cell lines displayed similar infectious IC_50_ values, with 95% CIs of 14–61 ng/ml for CHO and 13–21 ng/ml for pgsA-745. The result directly demonstrates that ι-carrageenan can disrupt steps in the HPV16 infectious pathway that do not involve HSPGs.

### PsV Inhibition by Consumer Products Containing Carrageenan

The widespread use of carrageenan in various consumer products led us to wonder whether lubricants intended for sexual use might employ carrageenan as a gelling agent. Internet searches revealed a number of sexual lubricant products that list carrageenan, or other algal polysaccharides, as ingredients. Although most lubricated condom brands (and some sexual lubricant brands) do not publicize their ingredients, one condom brand, Chapeau Crystal Carrageenan (Fuji Latex Co., Tokyo, Japan), advertises its use of a carrageenan-based lubricating gel [27].

Various sexual lubricant gels were subjected to testing in the HPV16 PsV inhibition assay. Several of the lubricant products were extremely potent inhibitors, with IC_50_ values occurring at dilutions of a few million–fold (Table 3). Two highly inhibitory European brands, Bioglide and Bioglide Anal, list carrageenan as an ingredient. The other components in these two products, water, glycerol, and xanthan gum, were noninhibitory when tested individually (Tables 2 and 3).

**Table 3:**
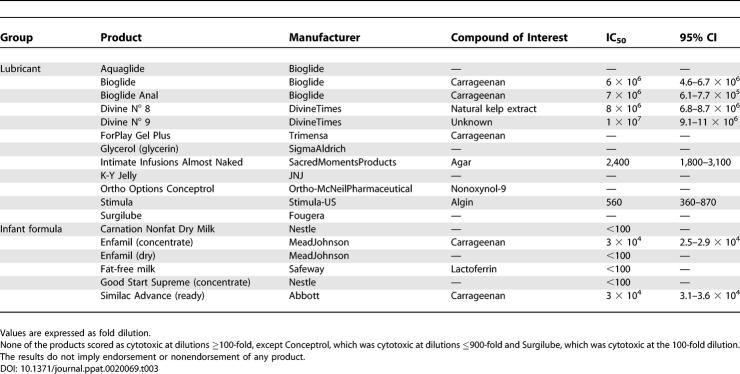
Inhibitory Effects of Consumer Products

One US product, Divine N° 8, uses the term “natural kelp extract” in its ingredient list. Although it is not clear what type of algal polysaccharide this term refers to, the high potency of Divine N° 8 in the HPV16 PsV inhibition assay strongly suggests that the product contains carrageenan. An unscented version of the product, Divine N° 9, does not list ingredients on its packaging or at its manufacturer's website, but its high potency suggests that it, too, may contain carrageenan. The inhibition curves for the Bioglide and Divine lubricants were similar to what would be expected if the products were composed of roughly 1%–3% carrageenan, a typical concentration range used to achieve gelation.

The packaging of a third US product, ForPlay Gel Plus, lists carrageenan as its fifth ingredient, behind several nonsulfated thickening agents. ForPlay Gel Plus did not display detectable inhibitory effects, even at dilutions as little as a hundred-fold. It is possible that ForPlay Gel Plus either contains very little carrageenan, or contains a less-inhibitory type of carrageenan. Alternatively, interactions between carrageenan and other compounds in ForPlay Gel Plus may abrogate the inhibitory effects of the carrageenan.

Lubricant brands containing other algal polysaccharides, such as agar and algin, were less effective for blocking the HPV16 PsV in the inhibition assay, consistent with the observation that these compounds are less inhibitory than carrageenan when tested individually (Table 2).

Several lubricant gels that do not contain sulfated polysaccharides were ineffective for blocking the HPV16 PsV at tested doses. Ortho Options Conceptrol, a contraceptive gel containing the detergent spermicide nonoxynol-9, does not contain sulfated polysaccharides. The fact that Conceptrol was ineffective for blocking the HPV16 PsV at noncytotoxic doses (Table 3) is consistent with a previous report demonstrating that nonoxynol-9 is not effective for blocking papillomavirus infectivity [28].

Another common use for carrageenan is as a stabilizing agent in milk-based products, including infant feeding formulas. The use of infant formulas containing carrageenan might thus be a factor in vertical transmission of HPVs, since such transmission could involve establishment of initial infection in infants' oral mucosa. We therefore tested several brands of infant formula using the HPV16 PsV inhibition assay. Formulas containing carrageenan displayed significant inhibitory effects, while fresh milk and infant formulas without carrageenan did not display detectable inhibitory effects at tested doses (Table 3).

## Discussion

In this report we demonstrate that carrageenan, an inexpensive commercial thickening agent extracted from seaweed, is an exceptionally potent inhibitor of papillomavirus infectivity in vitro. Carrageenan was found to be active against a range of common sexually transmitted HPV types that can cause cervical cancer and genital warts. Since carrageenan is generally recognized as safe for food and topical applications, it is an appealing candidate for use as a broad-spectrum topical microbicide to block HPV transmission.

Some, but not all, carrageenan-containing over-the-counter sexual lubricant gels we tested were extremely effective for blocking the infectivity of an HPV16 reporter pseudovirus in vitro. These results raise the possibility that use of such lubricant products, or condoms lubricated with carrageenan-based gels, could block the sexual transmission of HPV. However, in the absence of clinical efficacy data, it would be inappropriate to recommend currently available products for use as topical microbicides.

Carrageenan is also active in vitro and in murine model systems against other viruses, including herpes simplex viruses and some strains of HIV-1 [29–34]. However, in vitro IC_50_ values for carrageenan inhibition of herpes simplex virus and HIV-1 infectivity are about a thousand-fold higher than the IC_50_s we have observed for carrageenan inhibition of genital HPVs in vitro.

It is important to emphasize that cell culture systems may not fully represent some aspects of HPV infection of keratinocytes in vivo. However, our group has recently developed a pseudovirus-based murine genital challenge model for initial HPV infection (unpublished data). This animal model system should be useful for investigating of the potential efficacy of carrageenan for blocking HPV transmission in vivo.

A clinical trial focused on the effectiveness of a κ/λ-carrageenan preparation as a topical microbicide is currently in progress in South Africa. A recent patent application by the trial's organizers (http://www.popcouncil.org) contains a claim of carrageenan as a papillomavirus inhibitor, but the potency of the inhibitory effect was not indicated [35]. Since the principal focus of the ongoing trial is the efficacy of carrageenan against HIV-1, it may be necessary to develop additional clinical trials specifically focused on the in vivo efficacy of well-defined carrageenan preparations against HPVs. The high rate of acquisition of genital HPV infection in young adult populations (reviewed in [2]) might make it possible to perform short-duration clinical efficacy trials with relatively small numbers of human subjects.

Our results show that the principal mechanism by which carrageenan blocks papillomavirus infectivity is via the direct binding of carrageenan to the viral capsid. The binding of carrageenan appears to block interactions between the capsid and cell-surface HSPG attachment factors. Although the presence of HSPGs on the cell surface significantly enhances papillomavirus binding to and infection of most types of cultured cell lines [10,11,36,37], in this report we have used GAG-negative cells to demonstrate conclusively that HSPG attachment factors are not strictly required for infection to occur. A similar situation has been described for certain strains of HIV-1, particularly lab-adapted HIV-1 strains, for which HSPGs are thought to serve as attachment factors that facilitate (but are ultimately dispensable for) the in vitro infection of some cultured cell lines [38,39].

In addition to blocking the initial interaction between papillomavirus virions and HSPGs, carrageenan also exerts a second HSPG-independent inhibitory effect. This secondary inhibitory effect could be due to occlusion of virion surfaces involved in binding to cellular proteins involved in the infectious process. Alternatively, carrageenan might interfere with the development of needed conformational changes within the virion. Since it is possible that HPVs use alternative, non-HSPG attachment factors in vivo [36,37], the existence of a postattachment, GAG-independent inhibitory effect increases the likelihood that carrageenan might ultimately be effective as a topical microbicide against HPVs.

Although carrageenan was highly effective for neutralizing five different genital HPV types in vitro, it was substantially less potent against several papillomavirus types tropic for nongenital skin. Since common genital HPVs occupy a single genus, and the three nongenital papillomavirus types we have tested are phylogenetically distant from the genital types [22], it is tempting to speculate that all HPVs tropic for the genital mucosa would be comparably susceptible to inhibition by carrageenan. However, the possibility that some genital HPVs might exhibit natural resistance to inhibition by carrageenan would be an important factor to consider in the design of clinical efficacy trials.

Recurrent respiratory papillomatosis is a rare but debilitating HPV-induced condition involving the formation of large benign tumors on airway surfaces. The main treatment for the disorder is surgical removal of recurring obstructive masses. Juvenile onset recurrent respiratory papillomatosis (JORRP) is thought to be the result of vertical transmission of genital wart-associated HPV types 6 or 11 during birth (reviewed in [40]). The fact that some common infant formulas contain carrageenan raises the possibility that such formulas might function as inhibitors of the initial establishment of papillomavirus infection in newborns' oro-laryngeal epithelium [41]. Since human breast milk offers infants a wide variety of health benefits and JORRP is rare (roughly four cases per 100,000 births), it would be inappropriate to consider using infant formula for the purpose of preventing JORRP. However, the apparent safety of infant formulas containing carrageenan suggests that a pharmaceutical-grade carrageenan-based gel might be safe for perinatal cervico-vaginal application. The infrequency of JORRP, and its long initial latency period, would make it an impractical endpoint to use for clinical efficacy trials investigating the application of carrageenan-based gels. However, asymptomatic vertical transmission of genital HPV types, which is thought to be relatively common (reviewed in [42]), might be used as a surrogate endpoint that could readily be monitored by HPV DNA testing of infant buccal swabs.

## Materials and Methods

### Cell culture.

All cell lines were cultured in DMEM (Invitrogen, Carlsbad, California, United States) supplemented with 10% 56 °C heat-inactivated fetal calf serum (Hyclone, South Logan, Utah, United States), nonessential amino acids, and Glutamax-I (Invitrogen) (DMEM-10). CHO-K1 and pgsA-745 (American Type Culture Collection, Manassas, Virginia, United States) [26] were maintained in DMEM-10 supplemented with an additional 100 μM proline.

### Pseudovirus production.

Nucleotide maps of plasmids used in this work, as well as detailed protocols, are available at our laboratory website (http://home.ccr.cancer.gov/lco/default.asp). Various types of GFP-expressing pseudoviruses were produced according to previously described methods [7–9,43,44]. Briefly, 293TT cells were transfected with plasmids expressing the papillomavirus major and minor capsid proteins, L1 and L2, together with a GFP-expressing reporter plasmid, pfwB [8]. All PsV were produced using codon-modified L1 and L2 genes, except for HPV31 PsV, which used expression constructs based on wild-type L1 and L2 open reading frames. The high particle-to-infectivity ratio of HPV31 PsV stocks (Figure 4) is likely due to relatively poor expression of L2 (unpublished data). Codon-modified HPV45 L1 and L2 genes (p45L1w and p45L2w) were constructed based on sequencing of an HPV45 molecular clone. HPV16 PsV were produced using a previously unreported bicistronic L1/L2 expression plasmid, p16sheLL. Capsids were allowed to mature overnight in cell lysate, then purified using Optiprep gradients. The L1 protein content of PsV stocks was determined by comparison to bovine serum albumin standards in Coomassie-stained NuPAGE gels.

Fluorescently tagged capsids were generated by covalently conjugating Alexa Fluor 488 carboxylic acid, succinimidyl ester (Molecular Probes, Eugene, Oregon, United States) to HPV16 PsV, according to the manufacturer's instructions. Cell-binding inhibition results were also confirmed using fluorescent capsids generated by incorporation of an L2-GFP fusion protein [9]. Both types of fluorescent capsid displayed particle-to-infectivity ratios similar to wild-type HPV16 PsV (unpublished data).

### Inhibition assays.

Nonpolysaccharide candidate inhibitors were purchased from the suppliers shown in Table 1. Polysaccharides were purchased from Sigma-Aldrich (St. Louis, Missouri, United States), except for agarose (Invitrogen), agar (Difco, Detroit, Michigan, United States), and cellulose sulfate (Acros, Geel, Belgium).

Compounds were tested using a previously described PsV-based papillomavirus inhibition assay [9]. Briefly, HeLa cells were plated at 6,000 cells/well in 50 μl of medium in 96-well plates. Candidate inhibitors were dissolved at 1–10 mg/ml (or reconstituted as directed by manufacturer) in sterile water or appropriate solvent, then subjected to a ten-point three-fold serial dilution covering an appropriate concentration range. Diluted candidate inhibitor (50 μl) was added to preplated HeLa cells, followed by 50 μl of diluted PsV stock. The cells were incubated for 24 h, fed by addition of 100 μl of DMEM-10, then subjected to flow cytometric analysis 48–56 h after infection. IC_50_ values and 95% CI were determined using Prism (GraphPad Software, San Diego, California, United States) to calculate a variable slope sigmoidal dose-response curve.

PsV doses were calibrated such that between 5% and 20% of cells scored as GFP^+^ when no inhibitors were added. For HPV16, a standard dose with a final concentration of 1 ng/ml L1 (about 750 capsid equivalents of L1 per cell) resulted in fluorescence of about 10% of cells in the “no inhibitor” condition. Other papillomavirus types were used at the final L1 concentrations shown in Figure 4. HPV5 PsV infection of HeLa cells was performed with a dose of 35 ng/ml L1. Relative to HeLa cells, appropriate infection of HaCaT cells required a two- to three-fold higher capsid dose for HPVs 16, 18, and 45 and a two- to three-fold lower capsid dose for HPVs 5 and 6.

Cytotoxicity was defined as a >50% reduction in the net turnover of the metabolic substrate WST-1 (Roche, Indianapolis, Indiana, United States) and/or by the appearance of dramatic alterations in the microscopic appearance of cell morphology at the time of harvest.

The effect of acidic conditions on the inhibitory activity of ι-carrageenan was analyzed by inoculating HeLa cells with HPV16 PsV (1 ng/ml L1) together with various doses of ι-carrageenan in bicarbonate-free RPMI (Invitrogen) buffered to pH 7.4 with 10 mM HEPES, pH 5.0 with 10 mM acetic acid, or pH 4.5 with 10 mM lactic acid. The virus inoculum was removed after 2 h at 37 °C (ambient CO_2_) and replaced with DMEM-10 without carrageenan. Cell viability and PsV infectivity under acidic conditions were not significantly different from the neutral RPMI control.

Inhibition of capsid-to-cell binding was analyzed using HeLa and HaCaT cells detached using Cellstripper (Mediatech, Herndon, Virginia, United States), a proprietary, nonenzymatic chelating buffer. Suspended cells (3 × 10^4^ per condition) were incubated for 1 h at 37 °C with fluorescent capsids in a 150 μl volume of DMEM-10 supplemented various doses of ι-carrageenan. The cells were then washed and subjected to flow cytometric analysis. Binding IC_50_ was calculated based on net geometric mean fluorescence intensity using Prism software.

Time course analyses were performed in 96-well plates by incubating HPV16 PsV (1 ng/ml L1) with preplated HeLa cells for 2 h at 37 °C, followed by two washes to remove unbound virus. Various doses of carrageenan in DMEM-10 were then added to the cultures at the timepoints shown in Figure 5. For the 0-h timepoint, carrageenan was added to cultures immediately prior to virus inoculation, and a second carrageenan dose was added after washout of the inoculum. For the 2-h timepoint, carrageenan was added immediately after washout of the inoculum.

CHO-K1 and pgsA-745 cells were preplated overnight at 50,000 cells/well in 24-well plates. Cells were inoculated with 250 μl of HPV16 PsV at 45 ng/ml L1 (pgsA-745) or 0.9 ng/ml L1 (CHO-K1). The plates were incubated at 37 °C and gently swirled every 20 min for 3 h. The cells were then washed, and placed in 0.5 ml of medium containing various doses of carrageenan. The cultures were fed by addition of 2 ml of medium with no carrageenan after 24 h, and subjected to flow cytometric analysis 56 h after initial PsV inoculation.

### Capsid pull-down experiments.

Crosslinked 3% ι-carrageenan beads (Bioscience Beads, West Warwick, Rhode Island, United States) were used to perform HPV5 and HPV16 carrageenan direct-binding studies. HPV5 or HPV16 L1 (2 μg in the form of purified PsV) were incubated for 1 h at room temperature with 50 μl of carrageenan bead slurry pre-equilibrated into 1 ml of Dulbecco's PBS supplemented with 0.01% Tween-80 [45] and various concentrations of NaCl. BSA (4 μg) was used as a negative control. The beads were washed 3 × 10 min in Dulbecco's PBS containing appropriate concentrations of NaCl, followed by a final wash with plain Dulbecco's PBS. The beads were eluted by incubation at 65 °C in NuPage Load Dye (Invitrogen) with 8% 2-mercaptoethanol. Samples (10 μl out of about 75 μl total) were separated on NuPage gels (Invitrogen) and visualized using SYPRO Ruby Stain (Molecular Probes). Comparison of the samples to 1 μl of BenchMark Protein Ladder (Invitrogen) suggested an overall L1 recovery of roughly 75% in the 0.15 M NaCl condition (Figure 3).

## Supporting Information

### Accession Numbers

The GenBank (http://www.ncbi.nlm.nih.gov/Genbank) accession number for our sequencing of the L1 and L2 genes of an HPV45 molecular clone is DQ080002.

## Abbreviations

BPV1: bovine papillomavirus type 1
CHO: Chinese hamster ovary
GAG: glycosaminoglycan
GFP: green fluorescent protein
HPV: human papillomavirus
HSPG: heparan sulfate proteoglycan
IC_50_: 50% inhibitory concentration
JORRP: juvenile onset recurrent respiratory papillomatosis
PsV: pseudovirus
